# Ocular Rosacea Causing Corneal Melt in an African American Patient and a Hispanic Patient

**DOI:** 10.1155/2017/2834031

**Published:** 2017-10-11

**Authors:** Joanna S. Saade, Bachir Abiad, Jonathan Jan, Dana Saadeh, James P. McCulley, Jeremy Bartley

**Affiliations:** ^1^University of Texas Southwestern Medical Center, Ophthalmology Department, Dallas, TX, USA; ^2^American University of Beirut Medical Center, Dermatology Department, Beirut, Lebanon

## Abstract

**Purpose:**

To discuss two rare presentations of ocular rosacea in a Hispanic patient and an African American patient with unusual ocular manifestations.

**Case Report:**

Case  1: a 43-year-old Hispanic woman presented with right eye corneal perforation. Her prior medical history was significant for rosacea only, diagnosed clinically by a dermatologist. Her eye exam showed signs of bilateral ocular rosacea. An emergent full thickness tectonic corneal patch graft was done. The patient's bilateral eye symptoms improved one month after initiating rosacea treatment. Case  2: a 51-year-old African American man with long standing history of untreated rosacea presented with bilateral peripheral corneal thinning with neovascularization that led to right eye corneal perforation. Glue and bandage contact lens were applied. The patient did well 4 weeks after starting antibacterial, oral steroids, and rosacea treatment.

**Discussion:**

Ocular rosacea can present in Hispanic and African American patients with severe manifestations such as corneal perforation.

## 1. Rosacea

Rosacea is a chronic inflammatory cutaneous condition with a fluctuating course. It is characterized by flushing, nontransient erythema, papules, pustules, and telangiectasias. It is most commonly described in the white population of western and northern descent [1–3]. Although underestimated, it is also present in people with skin of color [4, 5]. Some reports state that 4% of rosacea patients are African American, Hispanic, or Asian [6, 7]. One study has shown that 2% of patients diagnosed with rosacea are African American and 3.5% are Hispanic [8].

Ocular manifestations have been reported in 3–58% of patients with rosacea [9–11]. They range from mild blepharoconjunctivitis with injection, tearing, and burning sensation to sight-threatening corneal neovascularization, scarring, thinning, and perforation [12].

Ocular rosacea was first described in African American patients in 1986 [13]. To our knowledge, there has not been a reported case of a Hispanic patient with corneal perforation secondary to rosacea. We present two cases of corneal perforation in patients with skin of color.

## 2. Case Report

### 2.1. Case  1

**Figure 1:**
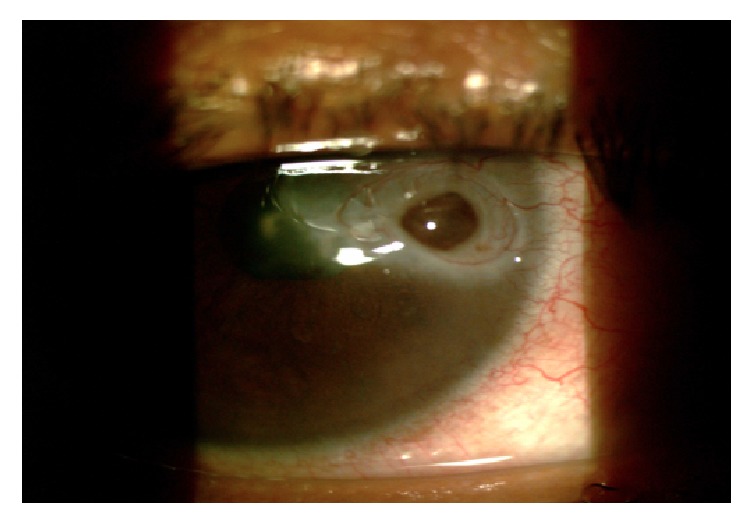
Slit lamp photo of the right eye showing the iris plugging the corneal perforation.

**Figure 2:**
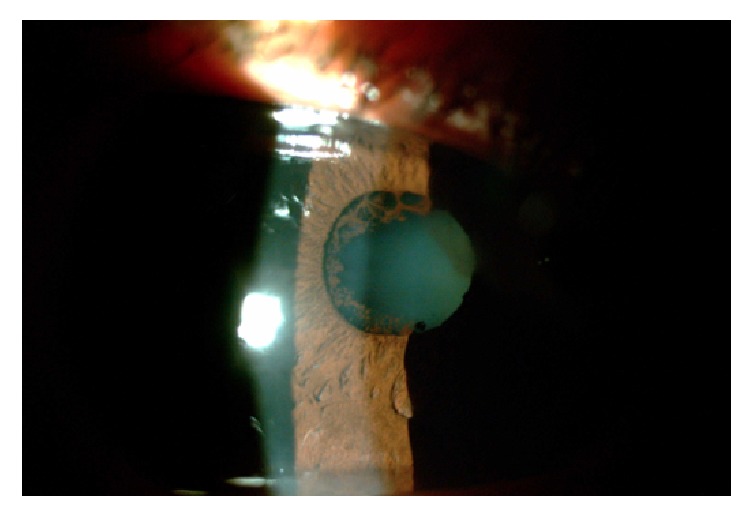
Slit lamp photo of the right eye showing a formed anterior chamber.

A 43-year-old Hispanic woman presented with recurrent asymmetrical bilateral eye redness, tearing, and pain for the past 2 years, with more severe symptoms in the right eye. Her ocular history was negative prior to this presentation. Her past medical history is significant only for rosacea, diagnosed clinically more than ten years ago by a dermatologist. She denied illicit drug use, exposure to patients with tuberculosis, or promiscuous sexual activity. Her family history is negative for ocular diseases and for autoimmune diseases. Aside from her eyes and her skin complaints, her review of systems was negative. Her right eye visual acuity was 20/40 and intraocular pressure was 8 mm Hg. Right eye slit lamp exam showed iris plugging a perforated cornea superonasally with associated extensive neovascularization (Figure 1) and a shallow but formed anterior chamber (Figure 2).

**Figure 3:**
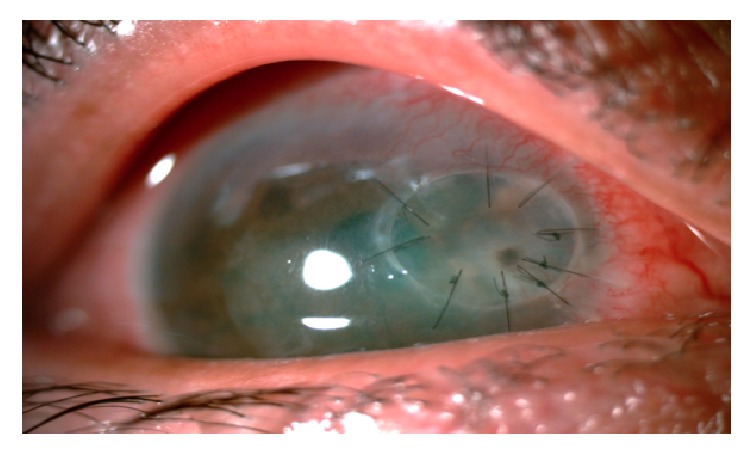
Slit lamp photo of the right eye showing a full thickness corneal patch graft, 1 week following the surgery.

The cornea in her left eye appeared normal. Signs of bilateral blepharitis due to ocular rosacea such as eyelid telangiectasias, thick meibomian gland secretions with digital compression, and collarettes on the base of upper and lower eyelashes were present. The patient consented to an emergent, full thickness, tectonic corneal patch graft. Postoperative management consisted of topical steroid (prednisolone acetate ophthalmic solution 1%) every two hours that was tapered slowly after one-week period and topical moxifloxacin four times daily for a total period of two weeks. Concurrently a dermatologist initiated rosacea treatment consisting of minocycline 100 mg orally twice daily and benzoyl peroxide/erythromycin gel daily. At week one after grafting, the visual acuity was 20/100 (Figure 3).

The patient's bilateral eye signs and symptoms improved significantly one month after initiating rosacea treatment.

### 2.2. Case  2

**Figure 4:**
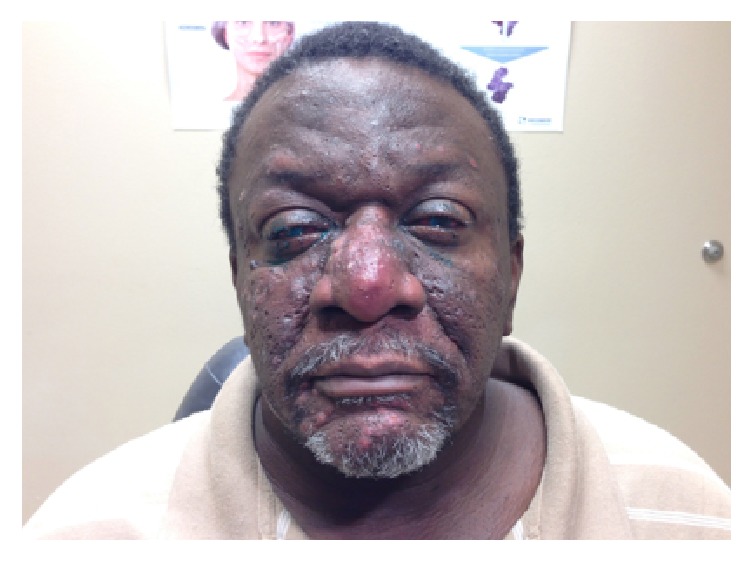
Rosacea manifesting with papules and pustules affecting the patient's face.

A 51-year-old African American man presented with bilateral eye pain and redness of four months' duration. Patient denied ocular problems previously. His prior medical history was significant for rosacea, for which he was not receiving any medical treatment. His review of systems was positive for bilateral eye pain and tender facial papules and pustules. He denied family history of autoimmune diseases or eye diseases. He denied promiscuous sexual activity, exposure to tuberculosis, or illicit drug use. His external exam was consistent with multiple medium to large erythematous papules, pustules with rhinophyma (Figure 4).

A skin biopsy was done and it revealed disrupted follicles with surrounding mixed suppurative and granulomatous dermatitis most suggestive of acneiform processes including ruptured folliculitis and rosacea. The patient was started on doxycycline 100 mg twice daily and topical metronidazole 1% cream twice daily.

His visual acuity was 20/400 in the right eye and 20/30 in the left eye. Slit lamp exam showed severe bilateral conjunctival injection, corneal pannus associated with inferonasal peripheral corneal thinning, and stromal scarring. His right eye showed an inferior 3 mm light-blocking infiltrate with overlying 3 mm epithelial defect.

**Figure 5:**
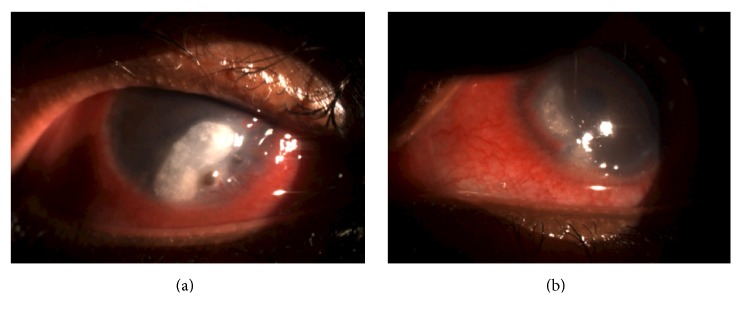
(a) Right eye slit lamp photo showing a large corneal infiltrate with a peripheral corneal perforation and pannus. (b) Left eye slit lamp photo showing peripheral ulcerative keratitis with scarring and pannus.

Peripheral ulcerative keratitis (PUK) was suspected and oral prednisone 60 mg was started. An extensive autoimmune workup was done. All results were negative. Cornea was scraped for gram stain, bacterial cultures, and fungal cultures. The patient was started on topical hourly fortified vancomycin (25 mcg/mL) and tobramycin (15 mcg/mL). The patient continued to worsen and within two weeks developed right eye inferior corneal perforation, for which adjacent conjunctival resection, glue, and a bandage contact lens were applied (Figure 5). Topical fortified vancomycin (25 mcg/mL) and tobramycin (15 mcg/mL) were given at a lower rate of six times daily. Oral treatment consisted of doxycycline 100 mg twice daily and 60 mg of prednisone for one week that was tapered by 20 mg on a weekly basis. Close follow-up showed slow improvement afterwards. After four weeks of treatment, the corneal infiltrate resolved. The patient provided his informed consent prior to publishing this information.

## 3. Discussion

Rosacea is a chronic inflammatory skin disorder with a yet unclear pathogenesis. The diagnosis of rosacea is usually a clinical one. Flushing, centrofacial erythema, and papules are most commonly observed. In 2002, the National Rosacea Society Expert Committee developed a classification system for rosacea, describing 4 different subtypes: erythematotelangiectatic, papulopustular, phymatous, and ocular rosacea [3]. Despite different treatment approaches, the committee agrees that there is overlap between the different subtypes. In patients with skin of color it may be difficult to perceive the signs of rosacea, particularly the erythematotelangiectatic subtype.

Additionally, ocular rosacea may be easily missed as 20% develop ocular manifestations before any skin lesions. Fifty-three percent of patients present with preceding skin findings and 27% present with both simultaneously [11].

The most common ocular manifestations involve the eyelids [14, 15]. In one report, 81% of patients presented with lid margin telangiectasia, 78% with meibomian gland dysfunction, and 65% with blepharitis [12].

Although less common, the more serious presentation involves the cornea. In one study, 33% of patients with ocular rosacea had corneal involvement [10]. The most common presentation was punctate epithelial erosions occurring in 13.6% [12, 14]. Wise reported that 67% of patients presenting with corneal complaints to the ophthalmology clinic had corneal neovessels and infiltrates [15].

Matrix metalloproteinases (MMP) are proinflammatory endopeptidases that have been implicated in corneal melting and stromal loss [16]. MMP-8 was found to be more elevated in the tear film of patients with rosacea compared with controls [17]. Multiple studies conducted by the Ocular Surface and Tear Center at Bascom Palmer Eye Institute looked at the concentrations of proinflammatory agents in the tear film of patients affected with rosacea and compared it to normal controls. In sum, they found that the concentrations of interleukin 1 alpha (IL-1 alpha), an inflammatory cytokine, gelatinase-B (pro-MMP-9), and MMP-9 were higher in the tear film of patients with rosacea as compared to controls [18, 19]. In particular, the concentration of pro-MMP-3 in the tear film of patients with corneal involvement due to rosacea was higher than the concentration found in controls and patients with rosacea without corneal involvement [20].

Studies looking at other proinflammatory agents in the skin of patients affected by rosacea found that there is excessive production of both cathelicidin (LL-37) and kallikrein (KLK5), the serine protease responsible for its cleavage [21–23]. In fact, LL-37 promotes inflammation, angiogenesis, and neovascularization [24]. Although not studied in the cornea of patients with rosacea, it can be hypothesized that if LL-37 was elevated in their tear film, being an angiogenic factor, it might be a contributor to corneal neovascularization. Understanding the pathogenesis of this disease may help in finding better treatment modalities.

The mainstay of treatment for blepharitis is lid hygiene [25]. Additionally, tetracycline in subantimicrobial levels has been proven to be effective in rosacea for many decades [26–28]. In particular, doxycycline and its derivatives were found to improve ocular signs and symptoms of rosacea [12, 29–35]. In fact, doxycycline 100 mg daily has been shown to improve ocular disease and increase the tear break-up time [36]. It was found to decrease the concentration of MMP-8 and MMP-9 in the tear film [17, 20]. Topical antibiotics have been used to alter the flora of the ocular surface in addition to topical steroids [34, 37].

In patients with corneal neovascularization and stromal loss, artificial tears and gel at night, topical cyclosporine, and cautious use of steroids are recommended in addition to the above-mentioned treatment modalities [38, 39]. Tissue adhesive or amniotic membrane or both should be applied over the cornea in the case of perforation or impending perforation [38, 40, 41]. Penetrating keratoplasty may be necessary in many severe cases [12, 41].

These two cases presented in the late stages of PUK due to rosacea with corneal perforation requiring interventions such as a tectonic patch graft and glue. Regardless of ethnicity, it is always important to maintain a high suspicion of rosacea in any individual with advanced corneal findings and negative laboratory tests.
